# Positron emission tomography of sodium glucose cotransport activity in high grade astrocytomas

**DOI:** 10.1007/s11060-018-2823-7

**Published:** 2018-03-10

**Authors:** Vladimir Kepe, Claudio Scafoglio, Jie Liu, William H. Yong, Marvin Bergsneider, Sung-Cheng Huang, Jorge R. Barrio, Ernest M. Wright

**Affiliations:** 1 Department of Molecular and Medical Pharmacology, The Geffen School of Medicine at UCLA, Los Angeles, CA 90095 USA; 2 Department of Nuclear Medicine, Cleveland Clinic, Cleveland, OH 44195 USA; 3 Department of Pathology and Laboratory Medicine, The Geffen School of Medicine at UCLA, Los Angeles, CA 90095 USA; 4 Department of Neurosurgery, The Geffen School of Medicine at UCLA, Los Angeles, CA 90095 USA; 5 Department of Physiology, The Geffen School of Medicine at UCLA, Los Angeles, CA 90095-1751 USA; 6 Present Address: Pulmonary and Critical Care Medicine, The Geffen School of Medicine at UCLA, Los Angeles, CA 90095 USA

**Keywords:** Astrocytomas, SGLT2, PET imaging

## Abstract

A novel glucose transporter, the sodium glucose cotransporter 2 (SGLT2), has been demonstrated to contribute to the demand for glucose by pancreatic and prostate tumors, and its functional activity has been imaged using a SGLT specific PET imaging probe, α-methyl-4-[F-18]fluoro-4-deoxy-d-glucopyaranoside (Me-4FDG). In this study, Me-4FDG PET was extended to evaluate patients with high-grade astrocytic tumors. Me-4FDG PET scans were performed in four patients diagnosed with WHO Grade III or IV astrocytomas and control subjects, and compared with 2-deoxy-2-[F-18]fluoro-d-glucose (2-FDG) PET and magnetic resonance imaging (MRI) of the same subjects. Immunocytochemistry was carried out on Grade IV astrocytomas to determine the cellular location of SGLT proteins within the tumors. Me-4FDG retention was pronounced in astrocytomas in dramatic contrast to the lack of uptake into the normal brain, resulting in a high signal-to-noise ratio. Macroscopically, the distribution of Me-4FDG within the tumors overlapped with that of 2-FDG uptake and tumor definition using contrast-enhanced MRI images. Microscopically, the SGLT2 protein was found to be expressed in neoplastic glioblastoma cells and endothelial cells of the proliferating microvasculature. This preliminary study shows that Me-4FDG is a highly sensitive probe for visualization of high-grade astrocytomas by PET. The distribution of Me-4FDG within tumors overlapped that for 2-FDG, but the absence of background brain Me-4FDG resulted in superior imaging sensitivity. Furthermore, the presence of SGLT2 protein in astrocytoma cells and the proliferating microvasculature may offer a novel therapy using the SGLT2 inhibitors already approved by the FDA to treat type 2 diabetes mellitus.

## Introduction

Cancer cells require high amounts of glucose as an energy source to grow and proliferate and this is the basis for positron emission tomography (PET) imaging with 2-deoxy-2-[F-18]fluoro-d-glucose (2-FDG) to detect and stage tumors. 2-FDG enters tumors via the facilitated glucose transporter GLUT1 (SLC2A1) where it accumulates following phosphorylation to 2-FDG-6-phosphate (2-FDG-6-P). In most cases, the high differential uptake of 2-FDG in cancer cells relative to that of surrounding tissues provides excellent imaging sensitivity.

For brain tumors, the level of 2-FDG-6-P accumulation depends on the density of GLUT1 transporters, the rate of hexokinase mediated 2-FDG phosphorylation, and the restricted efflux of 2-FDG-6-P from cells. In brain, the high rate of 2-FDG uptake in grey matter reduces tumor/background contrast and limits the utility of 2-FDG PET for imaging tumors.

In addition to the GLUT pathway for glucose uptake into cells, there is a second major class of glucose transporters known as the sodium glucose cotransporters (SGLTs or SLC5s) [1]. SGLT1 is expressed in the intestine and kidney, whereas SGLT2 is exclusively expressed in the kidney where it is responsible for glucose reabsorption from the glomerular filtrate. SGLT2 inhibitors, called glifozins, have gained recent clinical acceptance for the treatment of diabetes mellitus [2].

To assess the importance of this alternate glucose transport pathway in the body, we designed a new PET molecular imaging probe, α-methyl-4-[F-18]fluoro-4-deoxy-d-glucopyranoside (Me-4FDG) that is not a substrate for GLUTs [3]. The design of Me-4FDG was based on the knowledge that α-methyl-d-glucopyranoside is a non-metabolized substrate for SGLTs that is pumped into cells using the sodium concentration gradient across the cell membrane as a driving force.

We have previously reported on the importance of SGLT2 expression in pancreatic and prostate adenocarcinomas [4]. Here in a preliminary study, we report that SGLT2 is expressed in WHO Grade III and IV astrocytomas and that Me-4FDG PET provides a new high contrast metabolic imaging approach to detect and evaluate high-grade gliomas. This provides an entry into the understanding of the role SGLT-mediated glucose uptake pathway in astrocytoma growth and progression.

## Materials and methods

### Subjects

The preliminary study was performed, in compliance with guidelines set by the UCLA Institutional Review Board, on four adult brain tumor patients, and one adult with a history of epilepsy (Table 1). Three patients were newly diagnosed with WHO Grade IV astrocytomas (glioblastomas), and one newly diagnosed with a WHO Grade III (anaplastic) astrocytoma [5]. Their UCLA physician referred all patients and they, along with the healthy volunteers, gave their written informed consent. Table 1 summarizes the demographics, imaging and pathology findings of the four tumor patients, and the patient with a history of epilepsy, who for the purpose of this study is considered as a control subject. The tumor patients underwent clinical 2-FDG and MRI imaging (T1-weighted MP-RAGE with and without gadolinium contrast), and an experimental Me-4FDG PET scan 1 day prior to surgery. The epilepsy subject underwent clinical MRI, and interictal 2-FDG and Me-4FDG PET scans. Clinical evaluations of the 2-FDG and MRI scans in this subject were unremarkable with no localizing evidence for an epileptogenic focus.

**Table 1:** Patients in study

Patient	Age sex	MRI features	2-FDG PET	Surgical pathology
# 1	26 M	Unremarkable	Normal FDG uptake into cortical structures, basal ganglia, thalami, and cerebellum. No localizing evidence for epileptogenic focus in this patient with a history of epilepsy	N/A
#2	57 M	4.6 cm rim-enhancing mass in the posterior corpus callosum, with a mostly non-enhancing central portion. Second solidly enhancing 0.6 cm mass in the left frontoparietal white matter. White matter edema present	Large hypermetabolic mass in the bifrontal white matter with a hypometabolic central area. A smaller hypermetabolic focus is identified in the left frontoparietal white matter. No evidence of extracranial malignant disease	Glioblastoma (WHO Grade IV). Malignant fibrillary astrocytes, mitoses and extensive necrosis are present. Ki67 estimated at 35–45%
#3	70 M	5.3 cm mass in the paramedian left parietal lobe. Peripheral and solid enhancement. Necrotic non-enhancing area. Small amount of edema	Peripherally increased FDG activity with central photopenia	Glioblastoma (astrocytoma WHO Grade IV). Highly cellular infiltrating glial neoplasm with large areas of geographic necrosis. Numerous foci of glomeruloid endothelial hyperplasia and angiomatoid blood vessels are seen. Marked pleomorphism and hyperchromasia. Numerous mitoses are identified. Ki-67 estimated up to 15–20%
#4	67 F	2.7 cm mass in posterior left frontal lobe. Enhances heterogeneously	Area of increased FDG uptake in the left frontal lobe, posterior aspect. The body does not show any abnormal focus of FDG uptake	Glioblastoma (WHO Grade IV). Infiltrating glial neoplasm. Atypical glial cells with enlarged nuclei and some perinuclear halos suggestive of oligodendroglial differentiation. Mitoses are easily found. Areas of vascular endothelial hyperplasia. Pseudopalisading necrosis is present. Ki-67 estimated at 25–30%
#5	42 M	5.6 cm mass in the area of the left insula and anterior and mid temporal lobe with heterogeneous enhancement and significant surrounding vasogenic edema	Hypermetabolic activity in a mass in the left insula and left temporal lobe. No evidence for extracranial tumor	Gemistocytic anaplastic astrocytoma (WHO Grade III). Highly cellular, infiltrative GPAP positive glial tumor. Tumor cells show moderate to marked pleomorphism. Focal areas of punctate calcification are seen at the edge of the tumor, which also shows a minor oligodendroglial component. Multiple areas of robust glomeruloid endothelial proliferation. Rare mitotic figures are seen. Necrosis is absent. Ki-67 estimated focally up to 1–3%

As SGLT2 antibodies were not yet available at the time of the Me-4FDG PET studies, SGLT2 and SGLT1 immunochemistry (IHC) was subsequently carried out on 5 age-matched WHO Grade IV glioblastoma specimens selected from the UCLA Brain Tissue Translational Resource (Table 2). Suitable frozen archival sections were not available for the original PET patients. A board-certified neuropathologist (WHY) examined each slide used for immunohistochemistry (IHC) to confirm the presence of astrocytoma.

**Table 2:** SGLT expression in WHO Grade IV astrocytoma patient specimens as taken from a brain tumor tissue repository

ID	Diagnosis	Notes	Pathology in the IHC sample	SGLT1 staining	SGLT2 staining
#1	Gliosarcoma WHO Grade IV, recurrent; left occipital lobe		No tumor in the IHC sample; normal gray/white matter with reactive gliosis	Weak staining in neurons and/or astrocytes	Positive in reactive astrocytes + normal neurons in gray matter
#2	Glioblastoma, WHO Grade IV; left frontal lobe	EGFRVIII+	Diagnosis of glioblastoma confirmed; high cellularity, areas of necrosis and microvascular proliferation	Nuclear staining in 50% of cells, including tumor cells	Positive in tumor cells (cytoplasmic + nuclear staining) and in glia; positive in blood vessels
#3	Glioblastoma, WHO Grade IV; left temporal lobe		Diagnosis of glioblastoma confirmed	Nuclear staining	Cytoplasmic staining in tumor cells; also nuclear in some cells; blood vessels weakly positive
#4	Glioblastoma, WHO Grade IV; left frontal lobe	Ki67: 15–20% overall, 30% focally. GFAP diffusely positive	Diagnosis of glioblastoma confirmed; presence of abundant necrosis	Nuclear weak/moderate staining in 50–60% of the cells	Positive in tumor cells, with cytoplasmic + nuclear staining; some of the positive cells may be macrophages
#5	Glioblastoma, WHO Grade IV; right temporal lobe	Ki67: variable, overall 30%. GFAP diffusely positive. IDH1 R132H positive. MGMT methylation	Diagnosis of glioblastoma confirmed; presence of abundant necrosis	Nuclear moderate staining; some focal, very weak cytoplasmic signal is present	Extensive nuclear staining of moderate intensity; occasional cytoplasmic staining; some cells have a membrane pattern. A part of the tissue is normal brain, with positivity of SGLT2 in neurons (cytoplasmic)

### PET imaging

Me-4FDG was prepared as previously described from nucleophilic fluorination of its precursor methyl 2,3,6-tri-*O*-acetyl-4-*O*-triflyl-α-d-galactopyranoside using cyclotron-produced [F-18]fluoride [3]. The pure non-carrier added final product in saline solution and suitable for injection into humans had > 97% chemical and radiochemical purity and high specific activity (> 2000 Ci/mmol). All subjects received ~ 370 MBq (~ 10 mCi) of Me-4FDG (10.4 ± 1.6 µCi, range: 8.3–11.7 mCi) as a bolus injection via an indwelling venous catheter. Scans on astrocytoma and epilepsy patients (2-FDG and Me-4FDG) and healthy controls (Me-4FDG) were carried out on a Siemens/CTI ECAT Exact HR + PET or a Siemens Biograph PET/CT scanner.

Nine healthy volunteers without brain tumors or history of neurodegenerative disorders (7 males and 2 females aged 24–79) were recruited and underwent whole body Me-4FDG PET scans from the top of the head to mid-thigh. The first five scans were 10 min each, and the last three were 20 min each with total duration of 110 min. These scans were performed to determine bio-distribution of Me-4FDG in the brain and other organs over time.

Two tumor patients underwent brain PET scans after 30 min uptake time (consisting of four 300 s frames), and two were subjected to dynamic brain PET scans starting at the time of injection. In one case for 49 min (six 30 s frames, four 180 s frames, three 600 s frames and one 225 s frame), and the other case for 125 min (six 30 s frames, four 180 s frames, five 600 s frames, and three 1200 s frames).

The brain tumor patients and the epilepsy patient also underwent clinical 2-FDG brain PET scans as a component of their diagnostic work-ups, using a standard clinical protocol (180 MBq of 2-FDG, 40 min uptake time followed by 20 min head PET scan) [6].

Attenuation correction was performed using data from appropriate transmission scans using retractable [Ge-68]/[Ga-68] rods (ECAT HR + PET scanner) or CT imaging (PET/CT scanner). The acquired PET data covering a 15.5-cm in axial field of view was reconstructed using a filtered-backprojection method (after correction for attenuation, dead time, scatter and decay) with a Hanning filter (cutoff frequency, 0.3 cycle per projection element) into 128 × 128 × 63 matrices. The in-plane spatial resolution of the resulting brain images was ∼ 2.5 mm in full width at half maximum (5.5 mm for the whole-body scans).

Dynamic and static PET images were analyzed using the AMIDE software [7]. 3D regions of interest (ROI) were placed over the tumor, the torcula (confluence of sinuses) to determine blood activity, and on the hemisphere remote from the tumor covering both white and gray matter to determine the brain background (BG). As the Me-4FDG scans over remote areas from the tumor have a uniformly low signal, we cannot distinguish between grey and white matter. Values for all time points after 30 min within each ROI were summed and the resulting numbers for the tumor were divided by the summed number from the torcula ROI to give the SUVR, the tumor activity relative to venous blood, and the signal to noise (S/N) for the tumor relative to BG for white and grey matter. Tumor voxels with at least 90% of maximum value within the tumor ROI were used for calculation of the mean SUVR_peak_ value. Future dynamic studies will be conducted to extract the kinetic parameters of Me-4FDG uptake into tumors [8].

### Immunocytochemistry

SGLT2 is expressed in pancreatic and prostate adenocarcinomas [4], and so we reasoned that it may also be responsible for Me-4FDG uptake into brain tumors. The same polyclonal antibodies against SGLT2 (and SGLT1) were used to probe SGLT expression in the tumor bank frozen astrocytoma specimens. The SGLT antibodies did not recognize SGLT1 and SGLT2 in the paraffin sections retained from the tumors excised from the PET patients. The SGLT2 antibody was raised against a peptide corresponding to residues 591‒609 of human SGLT2, and the SGLT1 antibody was raised against a synthetic peptide corresponding to residues 563‒575 of human SGLT1. These antibodies were specific for SGLT2 and SGLT1 respectively as judged by Western Blotting and IHC on control tissues [4, 9–11]. The antibodies for the CD68 (mouse, DakoCytomation, M0876), GFAP (mouse, DakoCytomation, M0761), and CD163 (mouse, Cell Marque, 163M-16) markers were from commercial vendors; the dilutions used were 1/200, 1/200, and 1/50, respectively.

For the immunohistochemistry on 4 µm-thick frozen sections, the SGLT protocols were identical to those used recently for pancreatic and prostate tumors [4]. Antigen retrieval was performed: For CD68 by treatment in a pressure cooker in 0.05 M Tris (pH 9.0); for CD163 by treatment for 25 min in a vegetable steamer at 95 °C for 25 min in 0.001 M EDTA (pH 8.0, Invitrogen); and for GFAP by treatment for 25 min in a vegetable steamer at 95 °C in 10 mM citrate buffer (pH 6.0). Endogenous peroxidase was blocked by incubation with 0.3% H_2_O_2_ for 30 min, followed by washes in PBS and incubation for 30 min in blocking buffer (5% donkey serum, 0.1% NaN_3_ in PBS). Incubation with the antibodies with tissue sections was carried out overnight at 4 °C (SGLT2 and CD68) or at room temperature (SGLT1). For GFAP and CD163, the primary antibody was incubated for 1 h at room temperature. After washes, the samples were incubated with secondary antibody (Biotin-conjugated Donkey Anti-Rabbit antibody, Jackson Immunoresearch) for 90 min at room temperature and then with avidin/biotinylated enzyme complex (R.T.U. ABC Vectastain kit, Vector Laboratories) for 90 min at room temperature, followed by washes and incubation with the diaminobenzidine (DAB) substrate (Sigma) for 20 min. For CD163, CD68, and GFAP the secondary antibody (Envision System HRP-Labelled Polymer, anti-mouse, DakoCytomation) was incubated for 30 min, followed by DAB incubation for 5 min. Counterstaining was performed with diluted (1/5 in water) Harris hematoxylin (Sigma) and acid eosin. All microscopic slides were scanned with Aperio ScanScope AT scanner for analysis.

## Results

**Fig. 1:**
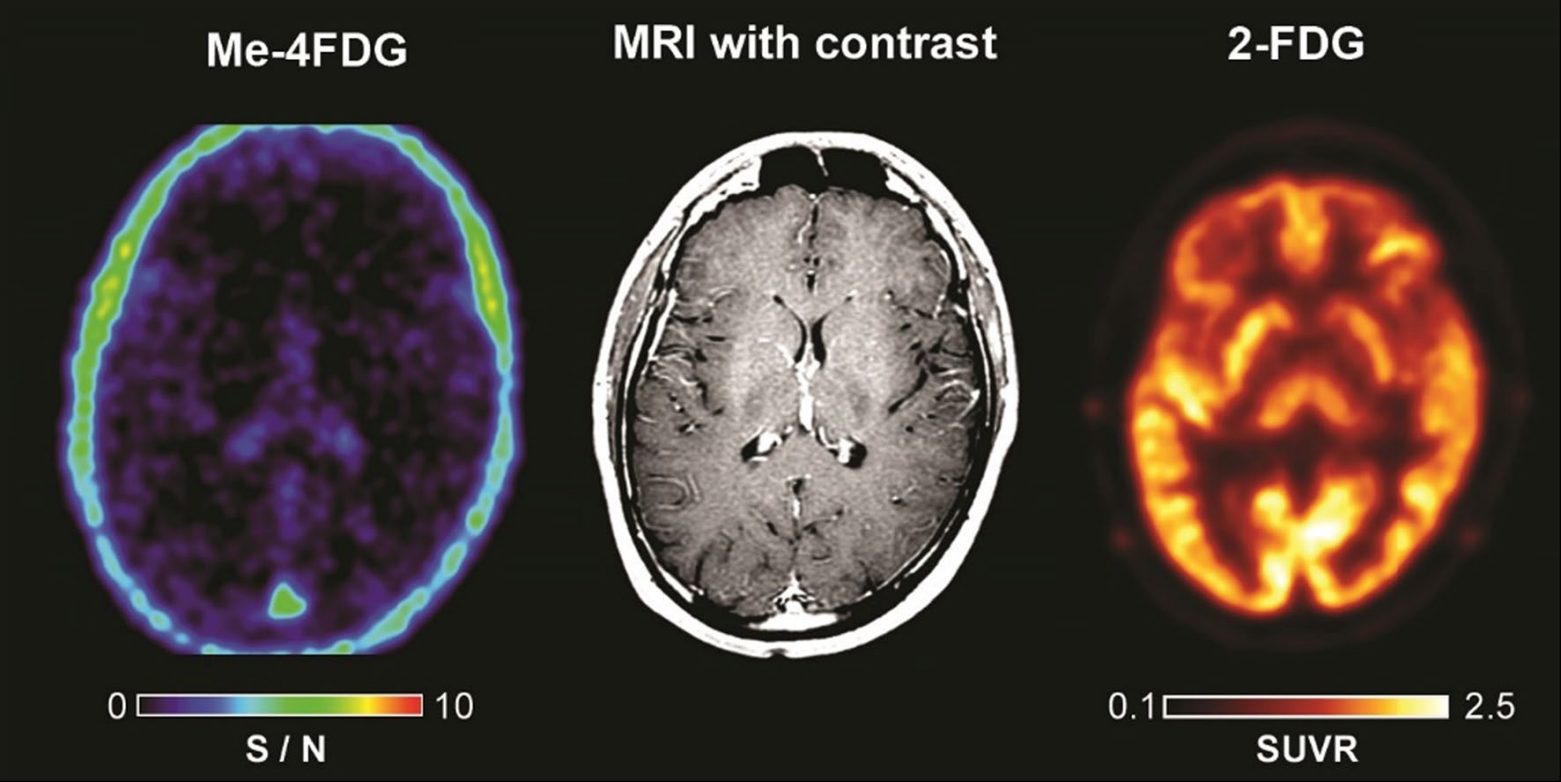
Me-4FDG, MRI, and 2-FDG PET scans on 26-year-old control subject. The Me-4FDG S/N (SUVR/BG) scale 0–10 is shown for the NIH color scale, and the 2-FDG SUVR scale is 0.1–2.5 for the Hot-Iron color scale

Representative co-registered trans-axial sections of the Me-4FDG, 2-FDG and contrast MRI scans on the control subject (diagnosed with epilepsy) are shown in Fig. 1. The subject did not have any seizures on the days of the scans and clinical evaluation of the 2-FDG scan was unremarkable with no localizing evidence for an epileptogenic focus. The accumulation of 2-FDG in the cortical gyri, subcortical nuclei, and cerebellum was read as normal. There was no significant uptake of Me-4FDG into any cortical or subcortical region of the brain with only a light Me-4FDG signal coinciding with the ventricles, sub-dural spaces, and the superior sagittal sinus. Similar Me-4FDG PET brain images were obtained for 9 healthy control human subjects (S/N 0.09 ± 0.02) and rodents [9, 10] demonstrating that SGLT transporters are not active in the blood-brain-barrier (BBB).

**Fig. 2:**
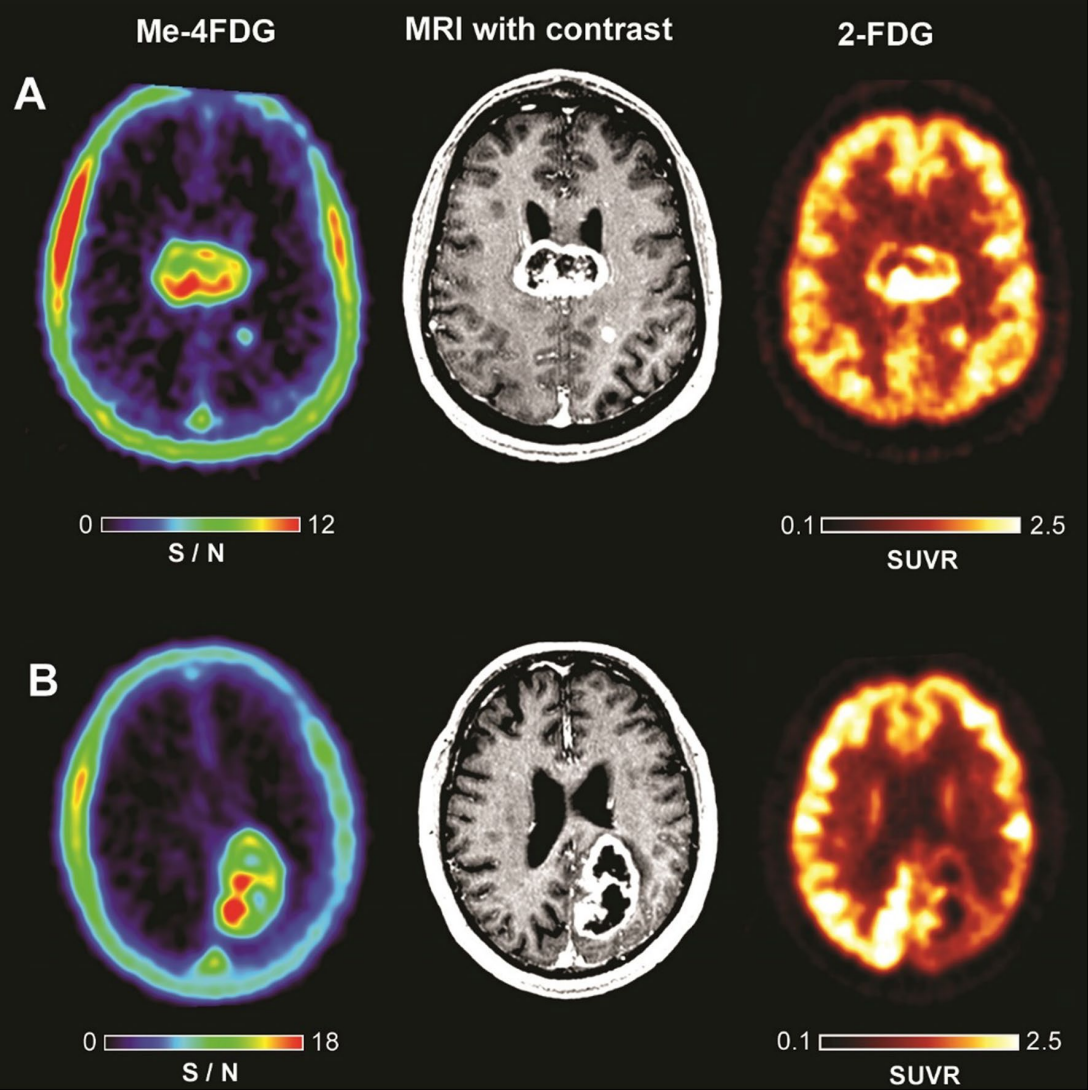
Me-4FDG PET, 2-FDG PET, and MRI scans on WHO Grade IV astrocytoma patients. **a** Patient with a 46-mm posterior corpus callosum astrocytoma, and **b** patient with 53-mm posterior para median left parietal lobe glioblastoma. For Me-4FDG the S/N (SUVR/BG) the NIH color scales are those relative to the torcula, and for 2-FDG the SUVR scales are for the “Hot-Iron” color scale. SUVR for 2-FDG was based on white matter as reference region and not blood. SUVR for Me-4FDG was based on blood activity and we re-normalized it to brain tissue so that we actually are comparing the same values. Thus, that is why S/N is specified for Me-4FDG and SUVR is specified for 2-FDG in the scale bar

Figure 2 illustrates representative co-registered Me-4FDG PET, MRI and 2-FDG PET brain scans from two patients with WHO Grade IV astrocytomas. The 57-year-old male patient (Fig. 2a) had a 46-mm enhancing mass in the posterior corpus callosum with a non-enhancing necrotic core. There was an additional 6 mm-solid enhancing mass in the left parietal white matter. Both the 2-FDG and Me-4FDG studies showed a high uptake of the respective tracers in both tumors largely corresponding to gadolinium uptake, but not the necrotic core of the larger tumor. There was normal 2-FDG activity, and no Me-4FDG activity throughout the remainder of the cortex, white matter, basal ganglia, thalamus and cerebellum. The PET scan shows that Me-4FDG was accumulated in the metabolically active regions of tumors. For the larger tumor, the Me-4FDG SUVR_peak_ value was 1.67 and the signal to noise ratio (S/N) was 11.5, and for the smaller mass SUVR_peak_ 0.84 and S/N 7.3. There was very low background signal elsewhere in the brain (S/N 0.09).

Figure 2b shows brain images of the 70-year-old male patient with a 53-mm peripherally enhancing glioblastoma mass in the left parietal lobe. The 2-FDG PET showed increased uptake in only the medial portion of the tumor, with the lateral gadolinium-enhancing portion having lower 2-FDG uptake than contralateral hemisphere gray matter. There was significant hypometabolism in the surrounding peritumoral cortex extending to the super left occipital lobe and left posterior cingulate gyrus (not shown). The regional uptake of Me-4FDG into the tumor closely resembles that for 2-FDG and the contrast MRI. The Me-4FDG SUVR_peak_ was 2.42 and S/N 16.7. From a clinical imaging perspective, the Me-4FDG-PET was superior to 2-FDG as in the latter most of the peripheral tumor uptake was equal to that of the surrounding gray matter. In both patients, Fig. 2a, b, there was high Me-4FDG uptake in the extracranial temporalis muscle [SUVR_peak_ 1.85 ± 0.55 (6)]. This is consistent with SGLT-mediated Me-4FDG uptake into muscles throughout the body [1].

**Fig. 3:**
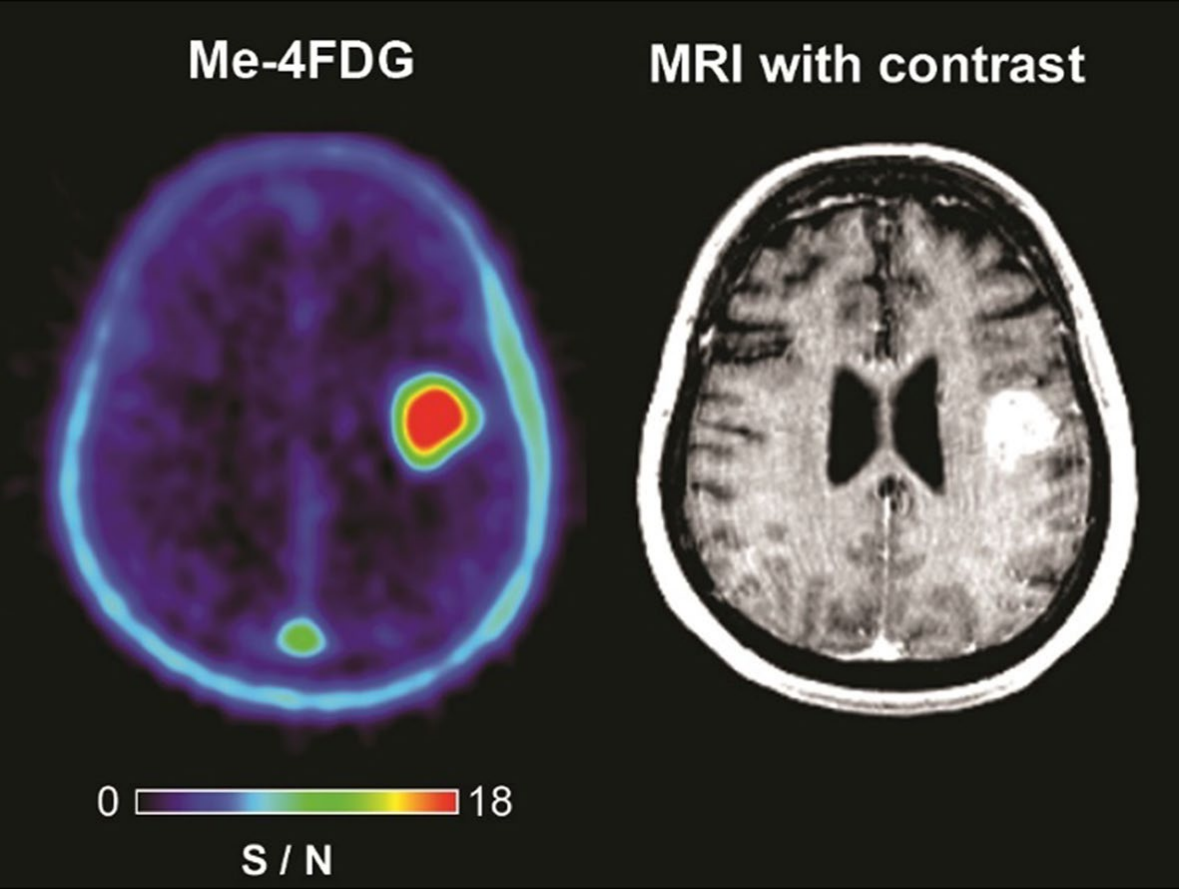
Me-4FDG PET and MRI scans on a WHO Grade IV astrocytoma patient. The Me-4FDG S/N (SUVR/BG) scale is for the NIH color scale

Figure 3 shows the Me-4FDG PET and MRI scans for a 67-year-old female patient with a 27 mm WHO Grade IV astrocytoma in the left posterior frontal lobe. The MRI scan indicates the presence of blood vessels within the mass, and the tissue pathology shows a branching vascular network with endothelial hyperplasia (not shown). 2-FDG PET demonstrated uniform accumulation in the tumor (not shown). Me-4FDG was uniformly accumulated in the mass to a level significantly higher than the surrounding grey and white matter. The Me-4FDG values were SUVR_peak_ was 1.57 and S/N 16.6.

**Fig. 4:**
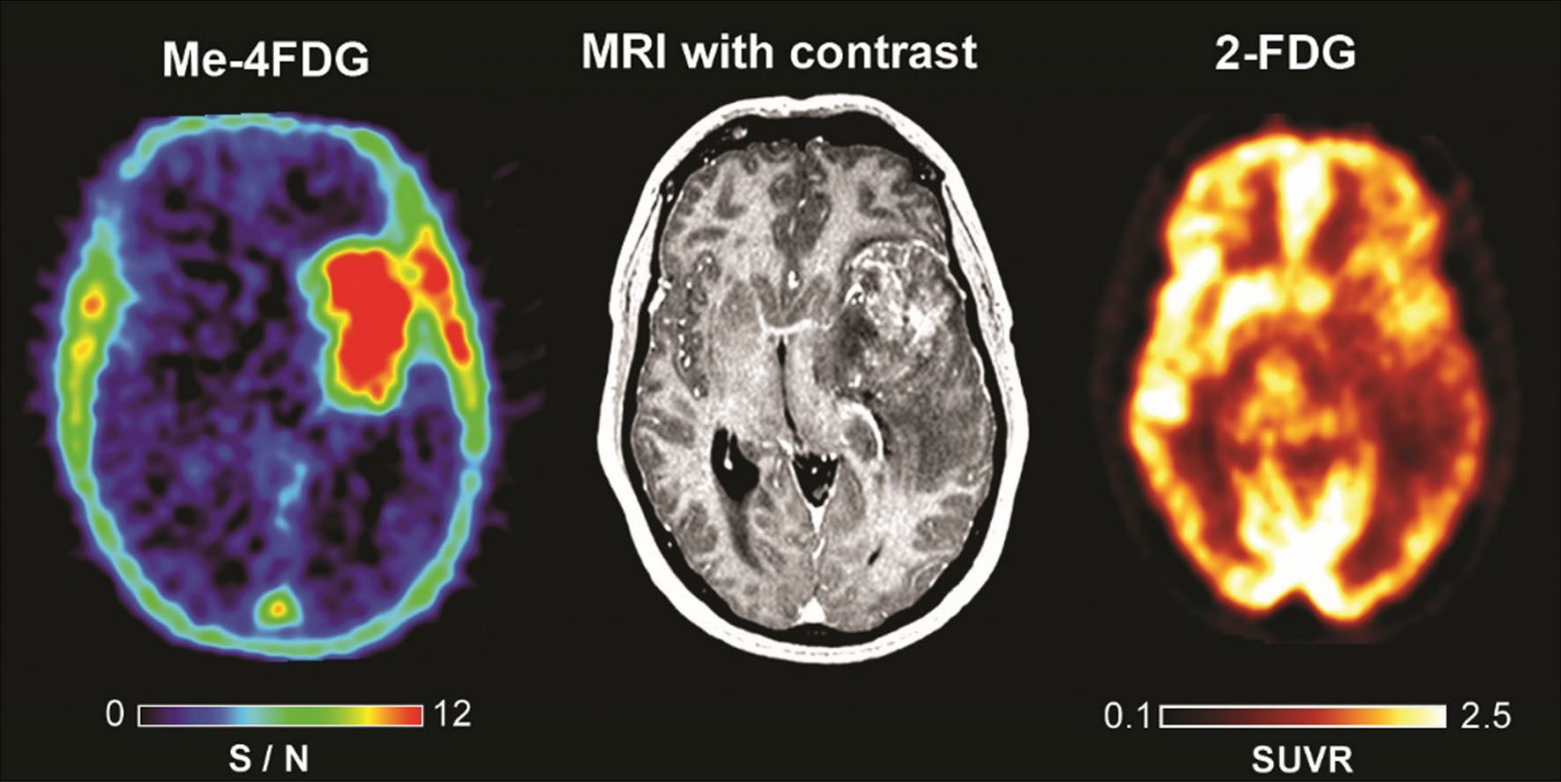
Me-4FDG PET, 2-FDG PET and MRI scans on a patient with an anaplastic astrocytoma (WHO Grade III). The Me-4FDG S/N (SUVR/BG) scales are for the NIH color scale and the 2-FDG SUVR scale is for the “Hot-Iron” color scale

Figure 4 shows the results of studies on a 42-year-old male patient with a heterogeneously enhancing 56 mm WHO Grade III astrocytoma. The tumor and surrounding edema caused significant mass effect and midline shift. On the 2-FDG PET scan, there was mixed uptake within some portions of the mass in the left insula and left temporal lobe. The highest tumor 2-FDG uptake was comparable to the normal uptake into the gyri of the right hemisphere and cerebellum. There was in contrast a uniform Me-4FDG uptake into the tumor (SUVR_peak_ was 2.55 and S/N 10.9) but none anywhere else in the brain (S/N 0.07). From a clinical diagnostic perspective, the 2-FDG PET was of minimal utility, whereas the Me-4FDG PET would have been far superior.

In the two cases with dynamic PET scans the uptake of Me-4FDG in the tumor exceeded that in venous blood (torcula) within 15–20 min and remained stable for the rest of the scan (data not shown). The SUVR (the ratio of activity in the tumor to that in venous blood) changed from 0.4 to 0.5 in the first 2 min to 1.27 and to 1.60 at 30 min. This demonstrates that the Me-4FDG within the tumor at 30 min comes from tumor uptake and only partially comes from the blood pool. The background value for the brain parenchyma (S/N ratio of activity in normal brain to torcula) was 0.09 ± 0.02 (n = 6), which is consistent with the nominal vascular volume in brain tissue. The S/N ratio obtained from the whole-body PET scans in the nine control subjects was higher, 0.32 ± 0.08, and this can be partially attributed to an underestimation of the torcula values.

### SGLT immunohistochemistry

**Fig. 5:**
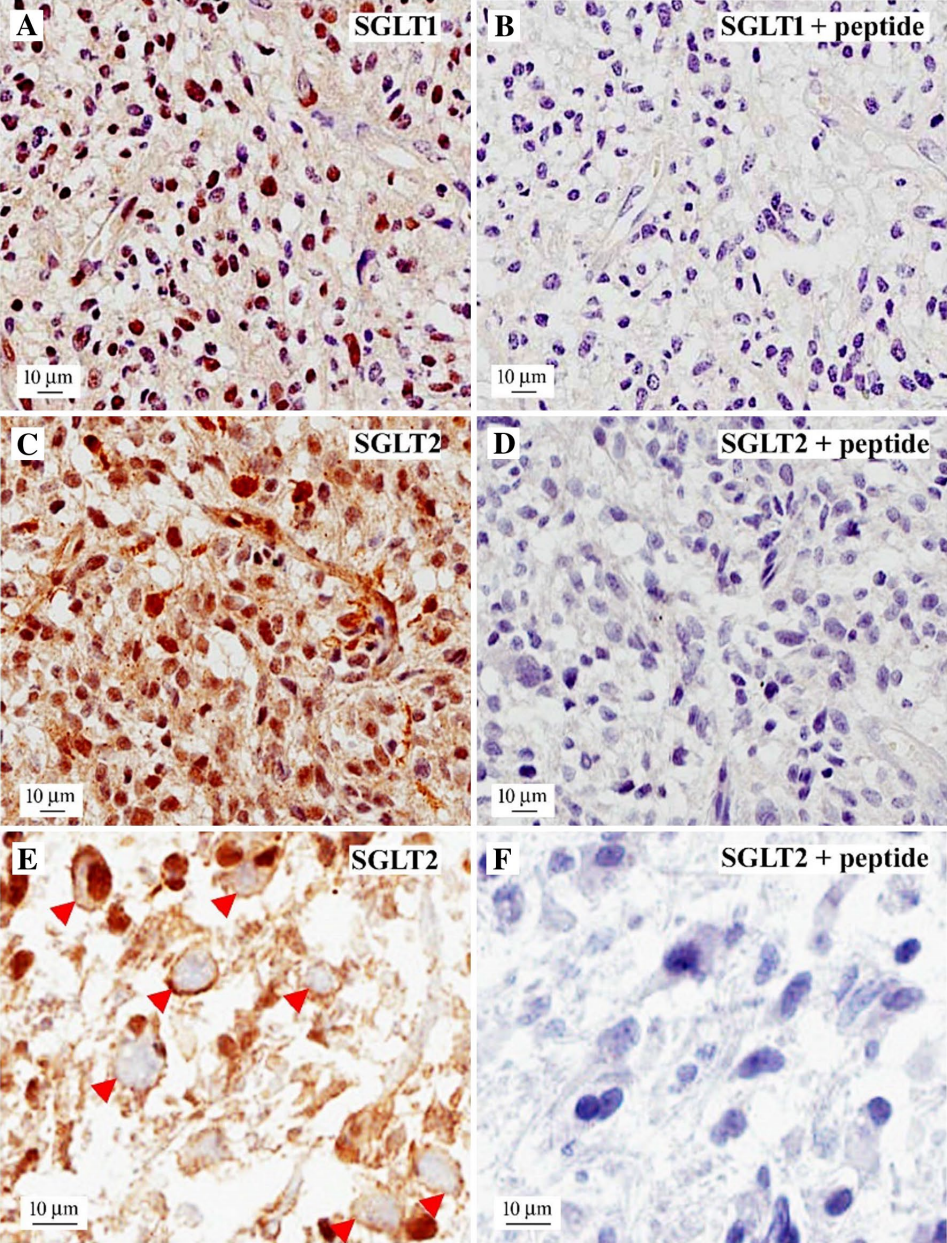
Expression of SGLT1 and SGLT2 in glioblastoma cells. A representative sample of human glioblastoma (WHO Grade IV) involving the left frontal lobe, typically characterized by high cellularity, was stained SGLT1, showing mostly nuclear signal (**a**); SGLT2, showing both nuclear and cytoplasmic staining in tumor cells (**c**). SGLT1 + antigenic peptide (**b**); and SGLT2 + antigenic peptide (**d**). A higher magnification image showing SGLT2 in or close to the plasma membrane (**e**, red arrowheads), and block by the antigenic peptide (**f**)

This was carried out on recent flash frozen specimens, as the antibodies did not recognize SGLTs in paraffin embedded normal kidney and in the glioblastoma specimens preserved from the PET patients. Figure 5 shows a typical case of a WHO Grade IV glioblastoma with high cellularity, with diffuse SGLT2 cellular staining in many neoplastic cells, but SGLT1 was restricted to the cell nuclei. The specificity of SGLT staining was confirmed in adjacent sections where the antibodies staining was blocked by the antigenic peptides used to raise the antibodies. At higher magnification (Panels E &F) specific SGLT2 labeling was evident in, or close to, the plasma membrane. At the resolution of light microscopy, it is not possible to resolve antibody staining between the cytoplasm and the cell membrane.

**Fig. 6:**
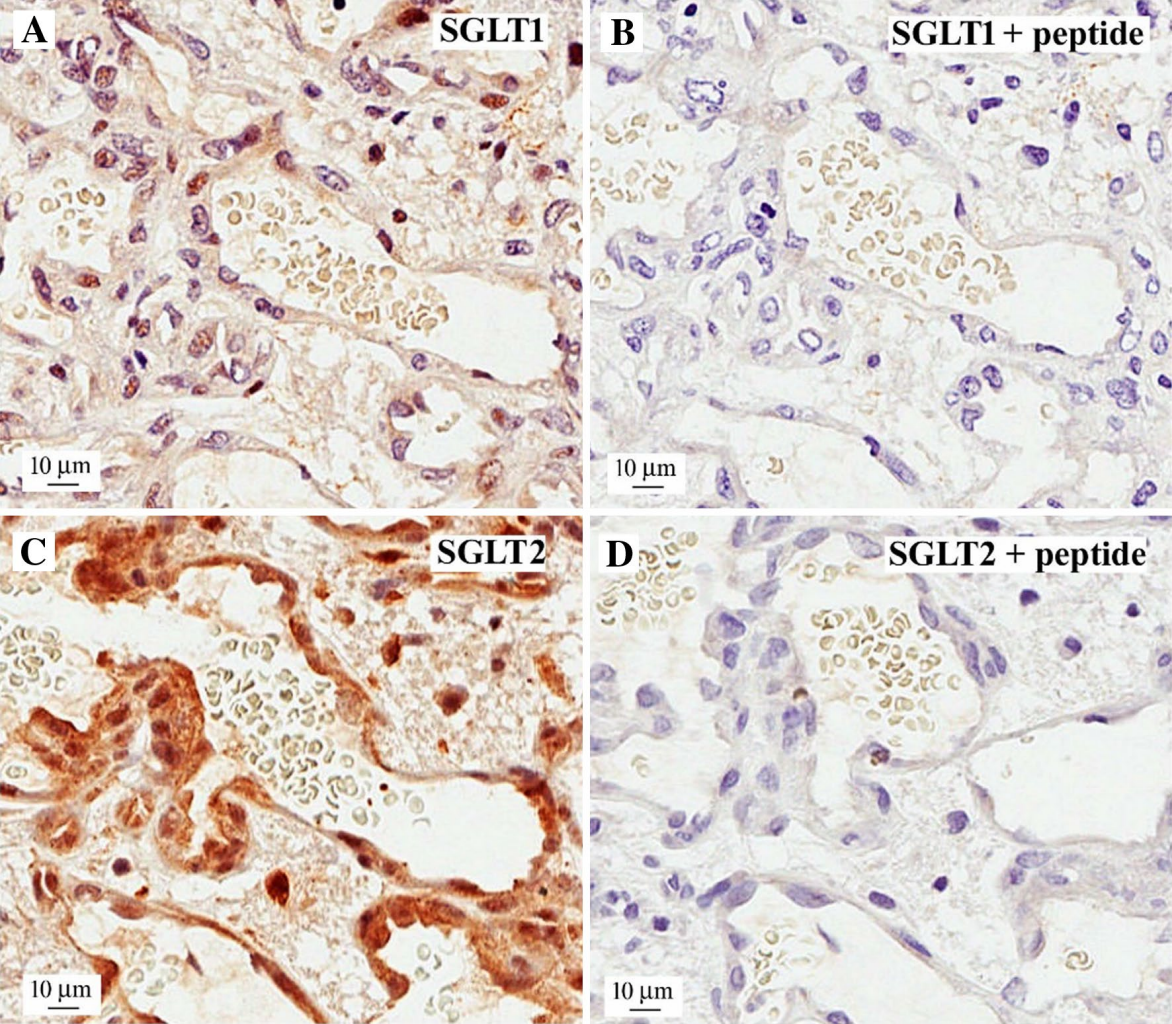
Expression of SGLT1 and SGLT2 in microvascular proliferation. A representative sample of human glioblastoma showing the characteristic, irregularly shaped microvascular proliferation stained with **a** SGLT1, **c** SGLT2 and **b, d** with appropriate antigenic peptides. Note the intense specific staining of the endothelial cells with the SGLT2 antibody

SGLT2 protein was also detected in the thin endothelium lining areas of tumor microvasculature (Fig. 6). This observation was surprising, because SGLTs are absent from normal brain capillary endothelium [9, 10], resulting in the lack of Me-4FDG penetration into the brain.

**Fig. 7:**
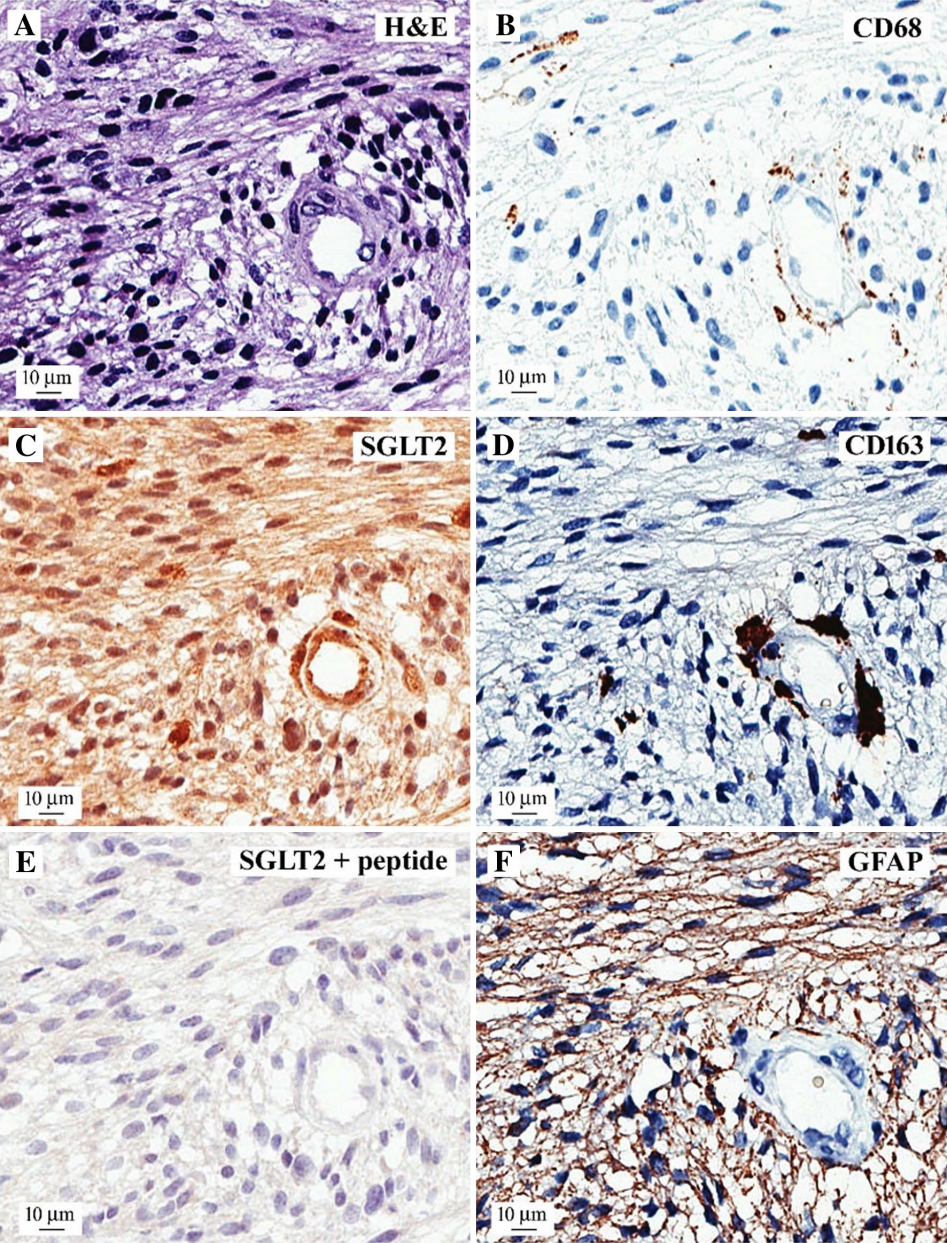
Relationship between SGLT2 expression and glial markers. Adjacent sections of a human glioblastoma sample were stained with different markers: **a** hematoxylin and eosin (H & E), showing high cellularity around a blood vessel; **b** SGLT2 expression in the vessel endothelium, in the microglia/macrophages surrounding the blood vessel, and the cancer cells; **c** negative control with SGLT2 antibody after pre-incubation with the antigenic peptide; and glial markers CD68 (**d**) and CD163 (**e**), showing restricted expression in microglia/macrophages surrounding the blood vessel, and GFAP (**f**), showing diffuse immune-positivity

We next wanted to exclude that SGLT2 was expressed only in glial cells infiltrating the diseased tissue; therefore, we stained adjacent sections with antibodies specific for microglia (CD68 and CD163) and for astrocytes (GFAP). Figure 7 presents a representative sample in which hematoxylin and eosin staining shows a WHO Grade IV glioblastoma with high cellularity (see also Fig. 5). CD68 and CD163 were positive in sparse cells, mostly in perivascular spaces, consistent with a microglial origin of the positive cells (Fig. 7d, e); both the endothelial cells and the perivascular microglial elements are positive for SGLT2 (Fig. 6c). The SGLT2 signal was specific, as it was completely abrogated by pre-incubation of the antibody with the antigenic peptide (Fig. 7c). Finally, GFAP shows diffuse positivity, consistent with the glial origin of the neoplastic cells. Taken together, these data showed that SGL2 is expressed in different elements of the tumor: malignant glioma cells, endothelial cells, and microglial cells.

No tumor was evident in the IHC slides provided for in one WHO Grade IV tumor sample (#1 Table 2). These contained normal gray and white matter with a reactive gliosis. There was weak specific SGLT1 staining in neurons and/or astrocytes. SGLT2 specific staining occurred in reactive astocytes and neurons, but none was evident for SGLT2 and SGLT1 in the microvessels.

## Discussion

This study introduces Me-4FDG PET as a novel, complementary, and in some respects superior clinical imaging modality for patients with high-grade astrocytomas. In each of the four high-grade astrocytoma patients imaged, the tumors avidly accumulated the Me-4FDG in stark contrast to the surrounding brain tissue. We readily acknowledge that, given the small study sample size, the potential clinical utility is not definable at this time, and further studies will be required to assess the utility of Me-4FDG PET in patients with molecular subtypes of glioblastoma, lower grade gliomas, and other glioma types.

In this preliminary study, the imaging characteristics visualized using Me-4FDG PET are superior to those of 2-FDG PET in brain tumors. Coupled with very low background levels of activity due to lack of SGLT expression on normal BBB, the observed Me-4FDG tumor activity levels were 11–17-fold higher than background and 1.6–2.6 fold higher than blood in these WHO Grade III and IV astrocytoma. The resolution of Me-4FDG PET can be gauged by the detection of a 6 mm mass in the left partial white matter of the patient with a WHO Grade IV tumor in the posterior corpus callosum (Fig. 2a). This provides evidence that 6 mm tumors may be detected by Me-4FDG PET in patients with other types of cancer, e.g. pancreatic adenocarcinomas.

Comparative studies will be required to assess the merits of Me-4FDG relative to other carbon-11 and fluorine-18 PET probes, such as amino acids (e.g. [C-11]methionine, [F-18]FDOPA, [F-18]FET, [F-18]fluciclovine [12–14]), or other targets expressed in glioblastomas (e.g. PSMA, CXCR4 [15–17]). Amino acid PET tracers exploit increased expression of amino acid transporters in tumor cells but their sensitivity is affected by the presence of amino acid transporters on normal BBB and other brain structures. The utility of PSMA or CXCR4 ligands is limited to WHO Grade IV glioblastomas that express their target proteins on their vasculature but not lower Grade astrocytomas that do not show such expression [18–20].

There are many questions that need to be addressed based on this exploratory study, including Me-4FDG pathobiology and tracer dynamics. The subjective co-analysis of MRI and Me-4FDG PET imaging revealed that the distribution of Me-4FDG uptake generally reflected the deposition of gadolinium; the latter generally considered reflecting an opening or loss of the BBB. Me-4FDG, unlike 2-FDG, does not cross the normal BBB in rodents and human subjects [9, 10] indicating that SGLTs are not functional in the endothelial plasma membranes of the normal BBB. The PET imaging in all cases confirmed that there was no entry of Me-4FDG into normal brain. Our demonstration of SGLT2 expression in endothelial cells in the proliferative tumor microvasculature suggests that the transporter contributes to Me-4FDG transport across the blood–tumor-barrier (BTB).

The results of our preliminary studies support the finding that the Me-4FDG within the tumor derives from a combination of tracer within tumor tissue and the blood pool. SGLT2 expression in neoplastic cells suggests that sodium symport contributes to Me-4FDG uptake within tumor cells. Unlike 2-FDG, Me-4FDG is a non-metabolized substrate for SGLT2 and accumulation of the tracer in cells is dependent on the density of SGLT2 proteins and the magnitude of the inward sodium electrochemical potential gradient [1]. Without the active transport of Me-4FDG into the tumor tissue one would expect similar activity levels in the tumor tissue and the blood pool due to broken blood-tumor barrier. Yet we observed an increase in tumor SUVR levels at later time points (beyond 15–20 min) when the blood radioactivity levels drop significantly.

How is Me-4FDG accumulated in glioblastomas? First, SGLT2 expression in endothelial cells of the proliferative microvasculature suggests that that this transporter may contribute to Me-4FDG transport across the BTB; and second, SGLT2 expression in neoplastic cells suggests that sodium symport contributes to Me-4FDG uptake.

There are two clinical implications of SGLT2 expression in glioblastomas. First, Me-4FDG PET/CT may be a useful imaging tool to detect brain tumors, even those as small as 6 mm, given the high signal to noise ratio signal for the brain. This is an obvious advantage over 2-FDG, as well as other carbon-11 and fluorine-18 PET imaging probes (see above). Second, SGLT2 inhibitors which have been developed to lower blood glucose levels in Type 2 diabetes mellitus may have a therapeutic potential for astrocytomas [2]. These drugs have a high affinity (K_i_ 1–5 nM) and specificity for SGLT2, e.g. the affinity of empagliflozin for SGLT2 is more than 2500-times higher than that for SGLT1.

It will be important to gain an understanding of what purpose the alternative SGLT2 mediated glucose uptake pathway, as opposed to the GLUT pathway, offers astrocyte tumors cells. If SGLT2 transporters on tumor cells provide for significant glucose uptake, then pharmacological SGLT2 blockage may have a starving effect and would disrupt their ability to grow and proliferate. In a preclinical trial SGLT2 inhibitors reduced the rate of growth and increased necrosis of a mouse model of pancreatic cancer [4].

We envisage that Me-4FDG PET may be useful in the future to image low-grade astrocytomas and other types of brain tumors, and may be able to follow the recurrence of tumors after therapy.

## Conclusions

A new glucose PET tracer, Me-4FDG, may provide highly sensitive imaging of high-grade astrocytomas. Me-4FDG, a specific substrate for SGLTs, does not cross the BBB and is not taken up by the normal brain. However, Me-4FDG crosses the BTB and is accumulated in tumors due to the expression of SGLT2 in proliferating microvessels and neoplastic cells. As a result, the signal to noise ratio for Me-4FDG uptake into tumors is superior to that for 2-FDG. SGLT2 is the new target for drugs to treat diabetes, and these may offer a novel therapeutic approach to treating brain tumors.
